# Previous infection with virulent strains of Newcastle disease virus reduces highly pathogenic avian influenza virus replication, disease, and mortality in chickens

**DOI:** 10.1186/s13567-015-0237-5

**Published:** 2015-09-23

**Authors:** Mar Costa-Hurtado, Claudio L. Afonso, Patti J. Miller, Eric Shepherd, Ra Mi Cha, Diane Smith, Erica Spackman, Darrell R. Kapczynski, David L. Suarez, David E. Swayne, Mary J. Pantin-Jackwood

**Affiliations:** Exotic and Emerging Avian Viral Diseases Unit, Southeast Poultry Research Laboratory, United States National Poultry Research Center, Agricultural Research Service, U.S. Department of Agriculture, Athens, GA USA

## Abstract

Highly pathogenic avian influenza virus (HPAIV) and Newcastle disease virus (NDV) are two of the most important viruses affecting poultry worldwide and produce co-infections especially in areas of the world where both viruses are endemic; but little is known about the interactions between these two viruses. The objective of this study was to determine if co-infection with NDV affects HPAIV replication in chickens. Only infections with virulent NDV strains (mesogenic Pigeon/1984 or velogenic CA/2002), and not a lentogenic NDV strain (LaSota), interfered with the replication of HPAIV A/chicken/Queretaro/14588-19/95 (H5N2) when the H5N2 was given at a high dose (10^6.9^ EID_50_) two days after the NDV inoculation, but despite this interference, mortality was still observed. However, chickens infected with the less virulent mesogenic NDV Pigeon/1984 strain three days prior to being infected with a lower dose (10^5.3–5.5^ EID_50_) of the same or a different HPAIV, A/chicken/Jalisco/CPA-12283-12/2012 (H7N3), had reduced HPAIV replication and increased survival rates. In conclusion, previous infection of chickens with virulent NDV strains can reduce HPAIV replication, and consequently disease and mortality. This interference depends on the titer of the viruses used, the virulence of the NDV, and the timing of the infections. The information obtained from these studies helps to understand the possible interactions and outcomes of infection (disease and virus shedding) when HPAIV and NDV co-infect chickens in the field.

**Electronic supplementary material:**

The online version of this article (doi:10.1186/s13567-015-0237-5) contains supplementary material, which is available to authorized users.

## Introduction

Avian influenza virus (AIV) and Newcastle disease virus (NDV) affect poultry worldwide and cause important economic losses [1]. Lower virulence viruses produce subclinical infections and occasionally upper respiratory disease and drops in egg production; however, more virulent forms of these viruses cause high mortality in poultry. AIV and NDV are single-stranded, negative-sense RNA viruses. AIVs are type A Orthomyxoviruses and are classified as low pathogenicity (LP) or high pathogenicity (HP) viruses based on their virulence in chickens and the presence of multiple basic amino acids at the cleavage site of the hemagglutinin precursor protein [2]. NDV’s, also known as avian Paramyxovirus type-1 viruses (APMV-1), are members of the genus *Avulavirus* in the Paramyxoviridae family. NDVs also vary in the type and severity of the disease they produce, and different pathotypes, based on their virulence in chickens and principally determined by the sequences surrounding the protease cleavage site of the fusion protein, have been described in poultry [3]. The original classification of NDV isolates into 1 of 3 virulence groups by chicken embryo inoculation as virulent (velogenic), moderately virulent (mesogenic), or as low virulence (lentogenic) has been recently modified for regulatory purposes. Velogenic and mesogenic viruses are now classified as virulent NDV, the cause of Newcastle disease (ND), whereas lentogenic viruses are the low virulence NDV widely used as vaccines [3]. The intracerebral pathogenicity index (ICPI) in day-old chicks is used to differentiate lentogenic viruses with ICPI values of less than 0.7, from virulent mesogenic strains with ICPI values equal to or greater than 0.7 and less than 1.5, and velogenic viruses with ICPI values greater than 1.5. Variant APMV-1 isolates from pigeons, referred to as pigeon paramyxovirus type 1(PPMV-1), by containing multiple amino acids in the fusion cleavage site are considered virulent NDV [3].

Virulent NDV isolates, the cause of ND, can spread rapidly leading to high mortality rates in poultry; the latter is an especially prominent feature of velogenic (*v*) NDV. In the US, and in many countries worldwide, ND is not endemic and prevention is focused on biosecurity and the vaccination of poultry with both live and inactivated NDV vaccines [3]. In order to control ND, intensive vaccination programs have been established in different countries around the world. The most widely used vaccines are formulated with NDV strains of low virulence, such as B1 and LaSota [3]. Some countries, usually those with endemic virulent NDV, use live mesogenic (*m*) NDV vaccines as booster vaccines [4-6]; but these *m*NDV are considered by the World Organization for Animal Health (OIE) to be virulent and therefore reportable for the purposes of trade if isolated from poultry [3]. HPAI is also a devastating disease for poultry and strategies for its control differ depending on the country, resources, subtype of the virus, and risk to public health. Eradication is the main goal, but has not been a realistic option for all countries, where vaccination may be considered as the only feasible option [7]. The current vaccines most commonly used are inactivated whole virus vaccines, but vectored vaccine use is increasing [8].

Little is known on the interactions between NDV and AIV when simultaneously infecting poultry. Co-infection of poultry with more than one etiologic agent is common and has resulted in increased clinical signs when compared to single agent infections [9-14]. Conversely, infection of a host with one virus may affect infection by a second virus, a phenomenon known as viral interference [15]. Exposure to NDV, either live vaccines or field strains, is almost unavoidable for commercial and non-commercial poultry worldwide, so co-infections with HPAIV are expected to occur in outbreaks and endemic situations. It is not clear if co-infections will exacerbate clinical signs of disease or if viral interference might occur and consequently mask or affect infections by one or other virus. AIV and NDV co-infections have been studied using cell cultures or chicken embryos, with interference between these viruses being reported, one virus inhibiting the growth of the other [16-19]; however, the mechanisms involved in such interference remains to be elucidated. In vivo studies examining AIV and NDV co-infections are scarce. A study using mallard ducks showed that co-infection with lentogenic (*l)* NDV and LPAIV did not affect the ability of ducks to become infected with either virus and had a minimal effect on viral shedding [20]. On the other hand, we found that co-infection of chickens and turkeys with a *l*NDV and a LPAIV affected the replication dynamics of these viruses, especially if given sequentially [21]. Similarly, a reduction of virus shedding and transmission was found in Pekin ducks co-infected with a *v*NDV and a LPAIV, and an increase in mean death time was observed in ducks infected with *v*NDV followed by a HPAIV compared to ducks infected only with HPAIV [22].

AIV’s are a continuous threat to poultry and public health worldwide. In order to control AI, it’s important to understand the pathogenesis of AIV’s in field conditions, and this includes co-infections with other viruses. The objective of this study was to determine if co-infection of chickens with NDV strains of different virulence could affect the outcome of infection with HPAIV’s. For this, we conducted experiments to examine the effect of simultaneous or sequential infection of chickens with lentogenic, and virulent (mesogenic and velogenic) NDV strains on HPAIV infections. Pathogenesis (clinical signs, lesions), presence of the viruses in tissues, and virus shedding were evaluated.

## Materials and methods

### Viruses

The following viruses were obtained from the Southeast Poultry Research Laboratory (SEPRL) repository: low virulent (*l*entogenic) NDV LaSota/1946 (vaccine strain, ICPI = 0.4), virulent (*m*esogenic) NDV Pigeon/1984 (ICPI = 1.45), virulent (*v*elogenic) NDV CA/2002 (ICPI = 1.85); and HPAIVs viruses A/chicken/Queretaro/14588-1988 (H5N2) and A/chicken/Jalisco/CPA-12283-12/2012 (H7N3). The viruses were propagated in specific pathogen free (SPF) embryonating chicken eggs (ECE), as previously described [23]. Virus-infected allantoic fluid was diluted in brain heart infusion (BHI) medium (BD Bioscience, Sparks, MD) in order to obtain an inoculum with titers of 10^4^ to 10^7^ 50% egg infectious dose (EID_50_) per 0.1 mL/bird. Sham inoculum was made using non-infected allantoic fluid from SPF ECE diluted 1:300 in brain heart infusion (BHI) medium (BD Bioscience, Sparks, MD, USA). The experiments were performed in biosecurity level-3 enhanced (BSL-3E) facilities in accordance with procedures approved by the SEPRL's Institutional Biosecurity Committee.

### Birds

Specific pathogen free (SPF) white leghorn chickens were obtained from SEPRL’s in-house flocks. The birds were housed in self-contained isolation units that were ventilated under negative pressure with inlet and exhaust HEPA-filtered air and maintained under continuous lighting. Feed and water were provided with *ad libitum* access. Birds were cared for in accordance to an SEPRL’s Institutional Animal Care and Use Committee approved animal use protocol.

### Experimental design

Three similar experiments were conducted. The first experiment examined the effect of simultaneous or previous inoculation of chickens with low virulence (lentogenic) (*l*), virulent (mesogenic) (*m*), and virulent (velogenic) (*v*) strains of NDV on HPAIV infection. A second experiment examined the effect of co-infection of chickens with the same *m*NDV and HPAIV strains, but the timing and dose of the HPAIV inoculation were modified. In order to corroborate the results of the second study, a third study further examined the effect of co-infection by using the same *m*NDV strain and a different HPAIV isolate in chickens at two different ages.

#### Study 1

Five-week-old SPF chickens were separated into a control group and virus-inoculated groups. The control group contained 12 birds, which were intraocularly (IO) (conjunctival sac of the right eye) and intranasally (IN) (choanal cleft) inoculated with 0.1 mL total of sham inoculum (group 1). The virus-inoculated groups, each also containing 12 birds, were inoculated IO and IN with 10^4.7–6.9^EID_50_ in 0.1 mL of the following viruses: group 2, *l*NDV strain: LaSota/1946 (10^6.9^EID_50_); group 3, *m*NDV strain: Pigeon/1984 (10^6.3^ EID_50_); groups 4 and 5, *v*NDV strain: CA/2002 (low and high dose: 10^4.7 or 6.3^ EID_50_); and group 6, HPAIV: A/chicken/Queretaro/14588-1988 H5N2 (10^6.9^ EID_50_). When birds were challenged with two viruses, both viruses were either administered at the same time (day 0; groups 7–10), or sequentially (HPAIV given 2 days after the NDVs; groups 11–14). Control groups were only exposed to one virus.

#### Study 2

Three groups of 12 3-week-old SPF chickens were inoculated IO and IN with the following: Group 1: 0.1 mL of sham inoculum given at day 0; Group 2: *m*NDV (Pigeon/1984) (10^6^ EID_50_ in 0.1 mL) given at day 0 followed by A/chicken/Queretaro/14588-1988 (H5N2) (10^5.3^ EID_50_ in 0.1 mL) given at day 3; and Group 3: A/chicken/Queretaro/14588-1988 H5N2 (10^5.3^ EID_50_ in 0.1 mL) given at day 3.

#### Study 3

Two and 4-week-old SPF chickens were separated into control groups and virus-inoculated groups. The control groups contained 6–8 birds, which were IO and IN inoculated with 0.1 mL of a sham inoculum (groups 1 and 6). The virus-inoculated groups, each containing 12 birds, were inoculated IO and IN with the following viruses: *m*NDV (Pigeon/1984) (10^6^ EID_50_ in 0.1 mL; groups 2 and 7) and HPAIV A/chicken/Jalisco/CPA-12283-12/2012 (H7N3) (10^5^ EID_50_ in 0.1 mL; groups 3 and 8). When birds were challenged with two viruses, the viruses were either administered at the same time (day 0; groups 4, 9) or sequentially (HPAIV given 3 days after the *m*NDV; groups 5, 10).

In all studies, birds were observed for clinical signs of disease over a 10-day period. Oropharyngeal (OP) and cloacal (CL) swabs were collected from all birds at days 1 and 2 (Study 1 and 2) or 1 through 7 (Study 3) days post-inoculation (dpi) to determine virus shedding. Two birds from each group in Studies 1 and 3 were euthanized at 2 dpi in single and simultaneously inoculated groups, and 2 days after HPAIV inoculation in sequentially infected groups. Gross lesions were recorded and tissues were collected in 10% neutral buffered formalin solution to evaluate microscopic lesions and the extent of virus replication in tissues by immunohistochemistry as described previously [24,25]. Portions of lung and spleen were also stored at −70°C for virus detection. Birds that stopped eating or drinking, had severe neurological signs, or remained recumbent were euthanized and counted dead as for the next day. Birds euthanized for necropsy, moribund birds, and all birds remaining at the end of the experiments were euthanized by the intravenous (IV) administration of sodium pentobarbital (100 mg/kg body weight).

### Virus titrations

OP and CL swabs were collected in 2 mL of BHI broth with a final concentration of 10 μg/mL of gentamicin, 100 units/mL of penicillin G, and 56 μg/mL of amphotericin B, and kept frozen at −70°C until processed. RNA was extracted using the MagMax AI/ND RNA isolation kit (Ambion, Inc. Austin TX, USA). Quantitative real time RRT-PCR (qRRT-PCR) for AIV and Newcastle disease virus (NDV) detection was performed as previously described [26,27] with modifications [21]. qRRT-PCR reactions targeting the influenza virus M gene [28] and the NDV M gene [29] were conducted using AgPath-ID one-step RT-PCR Kit (Ambion, Austin, TX) and the ABI 7500 Fast Real-Time PCR system (Applied Biosystem, Calsbad, CA). The RT step conditions for reactions were 10 min at 45°C and 95°C for 10 min. The cycling conditions for AIV were 45 cycles of 15 s, 95°C; 45 s, 60°C; and for NDV were 40 cycles of 10 s, 94°C; 30 s, 56°C; 10 s, 72°C. Virus titers in frozen lung and spleen samples were determined by weighing, homogenizing tissues, and diluting in BHI to a 10% (wt/vol) concentration. Equal amounts of RNA extracted from the tissue samples were used in the qRRT-PCR assay (50 ng/μL). For virus quantification, a standard curve was established with RNA extracted from dilutions of the same titrated stock of the challenge virus. Ct (cycle threshold) values of each viral dilution were plotted against viral titers. The resulting standard curve had a high correlation coefficient (r^2^ > 0.99), and it was used to convert Ct values to EID_50_. Results were reported as EID_50_/mL or EID_50_/g equivalents and the lower limit of detection was was 10^1.5^ EID_50_/mL for AIV and 10^1.7^ EID_50_/mL for NDV.

### Serology

In study 3, serology was conducted on serum from birds that survived. Hemagglutination inhibition (HI) assays were used to quantify antibody responses to virus infection as previously described [30]. Serum was collected from birds at 10 dpi (7 dpi from the second virus given in groups exposed to the viruses sequentially). Titers were calculated as the highest reciprocal serum dilution providing complete hemagglutination inhibition. Serum titers of Log_2_ 3 or lower were considered negative for antibodies against AIV or NDV.

### Statistical analyses

Data were analyzed using Prism v.5.01 software (GraphPad Software Inc. La Jolla, CA, USA). The survival rate data was analyzed using the Mantel-Cox Log-Rank test. Differences in the number of chickens positive for virus shed in co-infected groups compared to single-infected were analyzed with Fisher’s exact test (two-tailed). One-way ANOVA with Bonferroni multiple comparison analysis was used to evaluate virus titers in swabs. For statistical purposes, all OP and CL swabs and tissues from which virus was not detected were given a numeric value of 10^1.4^ EID_50_/mL for AIV and 10^1.6^ EID_50_/mL for NDV. These values represent the lowest detectable level of virus in these samples based on the methods used. Statistical significance was set at *P* < 0.05.

## Results

### Study 1

This study examined the effect of simultaneous or previous infection of 5-week-old chickens with *l*NDV, *m*NDV and *v*NDV on HPAIV infection.

#### Clinical signs and survival

**Figure 1:**
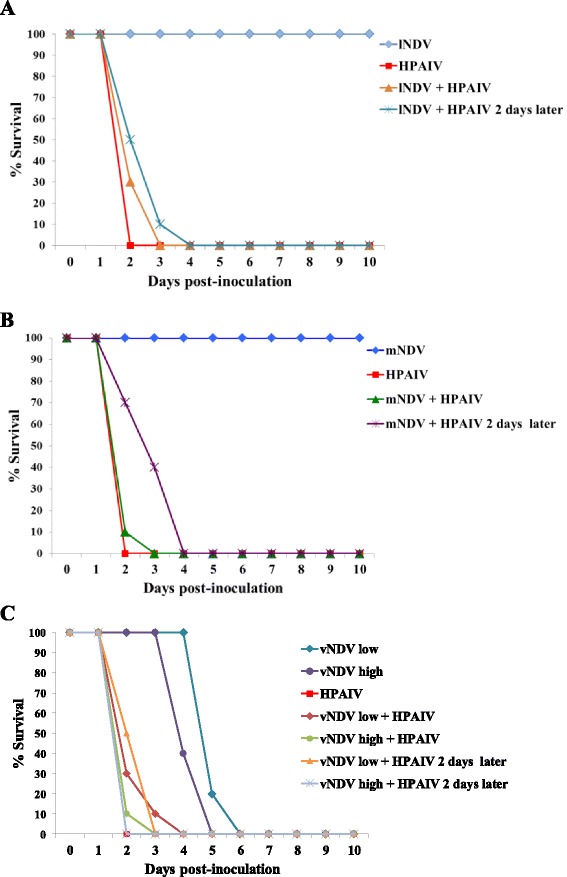
**Survival curves after inoculation of chickens with NDV and HPAI viruses (Study 1).** Chickens were inoculated simultaneously or sequentially with different strains of NDV and with HPAIV. Survival curves for chickens inoculated with *l*NDV and HPAIV (**A**), *m*NDV and HPAIV, and (**B**) *v*NDV and HPAIV (**C**).

None of the chickens inoculated with sham inoculum showed clinical signs. Chickens inoculated with *l*NDV and *m*NDV had mild conjunctivitis. All the *v*NDV and HPAIV-inoculated birds became severely sick and died with mean death times (MDT’s) between 1.9 and 5.2 days (Table 1 and Figure 1). Most of the birds died without previous overt clinical signs (peracute disease), but some showed non-specific clinical signs including conjunctivitis, ruffled feathers, lethargy, anorexia, swelling of the head and prostration, especially those birds infected only with *v*NDV, which also survived for longer. Some birds in the groups that received HPAIV also presented petechial-to-ecchymotic subcutaneous hemorrhages in leg shanks, feet and combs. No difference in the presence of clinical signs was observed between single-infected and co-infected birds.

**Table 1:** **Study 1: Mortality, mean death time (MDT), oropharyngeal (OP) and cloacal (CL) virus shedding in chickens inoculated with NDV and HPAIV**

**Virus**	**Number of dead/total (MDT)**	**OP shedding: number of birds shedding/total (Log**_**10**_ **EID**_**50**_ **/mL)** ^**a**^				**CL shedding: number of birds shedding/total (Log**_**10**_ **EID**_**50**_ **/mL)** ^**a**^			
		**NDV**		**HPAIV**		**NDV**		**HPAIV**	
		**1 dpi**	**2 dpi**	**1 dpi**	**2 dpi**	**1 dpi**	**2 dpi**	**1 dpi**	**2 dpi**
*l*NDV	0/10	10/10 (5.3 ± 0.1)^A^	10/10 (6.1 ± 0.1)^A^	nd	nd	1/10 (1.6)^A^	5/10 (3.3 ± 0.08)^A^	nd	nd
*m*NDV	0/10	7/10 (2.9 ± 0.4)^A^	10/10 (3.2 ± 0.1)^A^	nd	nd	0/10 (0)^A^	0/10^A^	nd	nd
*v*NDV low dose	10/10 (5.2)	10/10 (4.4 ± 0.1)^A^	10/10 (5.9 ± 0.1)^A^	nd	nd	0/10 (0)^A^	8/10 (3.8 ± 0.2)^A^	nd	nd
*v*NDV high dose	10/10 (4.4)	10/10 (3.6 ± 0.1)^A^	10/10 (6.4 ± 0.1)^A^	nd	nd	0/10 (0)^A^	10/10 (5.2 ± 0.1)^A^	nd	nd
HPAIV	10/10 (2.0)	nd	nd	10/10 (4.6 ± 0.1)^A^	2/2 (5.7 ± 0.1)^A^	nd	nd	8/10 (3.1 ± 0.3)^A^	2/2 (4.9 ± 0.3)^A^
*l*NDV + HPAIV	10/10 (2.3)	9/10 (4 ± 0.1)^B^	5/10 (4.5 ± 0.1)*^B^	9/10 (4.6 ± 0.1)^A^	5/5 (6 ± 0.1)^A^	0/10 (0)^A^	0/5^B^	8/10 (2.7 ± 0.3)^A^	5/5 5.6 ± 0.2)^B^
*m*NDV + HPAIV	10/10 (2.2)	2/10 (1.8 ± 0.04)*^A^	1/3 (2.2)*^B^	10/10 (4.2 ± 0.1)^A^	3/3 (5.9 ± 0.3)^A^	0/10 (0)^A^	0/3^A^	8/10 (2.4 ± 0.2)^A^	3/3 (5.4 ± 0.03)^A^
*v*NDV low dose + HPAIV	10/10 (2.4)	5/10 (4.2 ± 0.2)*^A^	5/5 (4.7 ± 0.1)^B^	10/10 (4.5 ± 0.1)^A^	5/5 (5.8 ± 0.2)^A^	0/10 (0)^A^	0/5**^B^	7/10 (2.5 ± 0.4)^A^	5/5 (5.5 ± 0.2)^A^
*v*NDV high dose + HPAIV	10/10 (2.1)	10/10 (4.6 ± 0.3)^B^	3/3 (6.3 ± 0.1)^A^	10/10 (4.3 ± 0.1)^A^	3/3 (6.4 ± 0.3)^A^	0/10 (0)^A^	0/3**^B^	7/10 (2.5 ± 0.4)^A^	3/3 (5.5 ± 0.2)^A^
*l*NDV + HPAIV 2 days later	10/10 (2.6)	10/10 (3.9 ± 0.1)^A^	7/7 (6.6 ± 0.2)^A^	10/10 (4.5 ± 0.2)^A^	7/7 (5.9 ± 0.2)^A^	2/10 (2.1 ± 0.01)^A^	0/7*^A^	7/10 (2.3 ± 0.4)^A^	7/7 (5.3 ± 0.04)^A^
*m*NDV + HPAIV 2 days later	10/10 (3.1)	7/10 (2.4 ± 0.2)^A^	7/9 (3.1 ± 0.1)^A^	10/10 (3.8 ± 0.2)^A^	9/9 (5.8 ± 0.1)^A^	4/10 (1.8 ± 0.1)^B^	5/9 (3.2 ± 0.2)*^B^	10/10 (3.1)^A^	9/9 (5.2 ± 0.04)^A^
*v*NDV low + HPAIV 2 days later	10/10 (2.5)	10/10 (6.4 ± 0.1)^B^	7/7 (7.6 ± 0.1)^B^	3/10 (1.5 ± 0.7)**^B^	7/7 (5.3 ± 0.1)^A^	10/10 (5.5 ± 0.1)***^B^	7/7 (7.1 ± 0.1)^B^	0/10***^B^	7/7 (5.3 ± 0.05)^A^
*v*NDV high + HPAIV 2 days later	10/10 (1.9)	10/10 (6.8 ± 0.1)^B^	2/2 (8.0 ± 0.3)^B^	3/10 (2.1 ± 0.1)**^B^	2/2 (5.3 ± 0.1)^A^	10/10 (6.3 ± 0.1)***^B^	2/2 (7.6 ± 0.3)^B^	0/10***^B^	2/2 (5.3 ± 0.05)^A^

For groups inoculated sequentially with the viruses, HPAIV titers are from 1 or 2 days after HPAIV inoculation, which corresponds to days 3 and 4 after NDV inoculation.

^a^Viral titers average ± SEM (standard error mean) from the positive birds; Log_10_ EID_50_ equivalents were determined by qRRT-PCR. nd = not done. ^*^Significant difference in number of chickens virus positive compared to single virus infected groups (*P < 0.01; **P < 0.001; ***P < 0.0001).

^A;B^Different superscript uppercase denote significant difference in virus titers compared to single virus-infected groups (*P* < 0.05).

Bird survival was compared among groups after single or simultaneous inoculation of the viruses, and after inoculation with HPAIV in groups sequentially infected with the viruses. All birds inoculated only with *l*NDV or *m*NDV, survived and showed significant differences in survival when compared with the rest of the groups. Differences in survival were also found between single-infected *v*NDV groups (CA/2002) depending on the dose, 10^4.7^or 10^6.3^ EID50, (*P* < 0.01); MDT was lower with the higher dose (MDT’s of 4.4 days vs. 5.2 days).

Differences in survival were observed between groups infected only with the *v*NDV (low or high dose) and birds inoculated simultaneously or 2 days later with the HPAIV (*P* < 0.0001). Low dose of *v*NDV increased the survival of the birds when given HPAIV either simultaneously or sequentially, when compared to the group inoculated only with HPAIV (*P* < 0.05), contrary to the high dose *v*NDV which did not have that effect.

Simultaneous co-infection with *l*NDV or *m*NDV and HPAIV did not significantly increase the survival of the birds; however, a significant difference in mean death time was found when comparing birds sequentially infected with these viruses and HPAIV and birds that received only HPAIV (*P* < 0.01), MDT’s of 2.6 and 3.1 versus 2.0 days.

#### Viral shedding

OP and CL viral shedding were examined at 1 and 2 dpi by qRRT-PCR and results are shown in Table 1. To compare the effect of co-infection with NDV on HPAIV replication, birds sequentially inoculated with NDV and HPAIV were evaluated at days 3 and 4 after NDV inoculation), corresponding to days 1 and 2 after HPAIV inoculation.

NDV was detected in most OP swabs from birds inoculated only with NDV (*l, m or v*) or from birds sequentially inoculated with the HPAIV, these later groups corresponding to 3 and 4 days after NDV inoculation. Fewer birds were positive for *l*NDV at 2 dpi (OP and CL swabs) (*P <* 0.01), and lower OP NDV titers (*P <* 0.05) at 1 and 2 dpi, were observed from the group inoculated simultaneously with HPAIV when compared to the *l*NDV single-inoculated group. Similarly, lower numbers of birds shed *m*NDV or *v*NDV (low dose) by the OP route (*P <* 0.01) and lower viral titers (*P <* 0.05) at 2 dpi, were observed from the groups inoculated simultaneously with HPAIV when compared to the single-inoculated groups. None of the birds inoculated simultaneously with the *v*NDV (low dose) and HPAIV shed *v*NDV by the CL route at 2 dpi, different to the NDV single-inoculated group where 8 of 10 birds shed virus at this time point. All birds simultaneously inoculated with the high-dose *v*NDV and HPAIV shed virus by the OP route at 1 and 2 dpi, and higher OP titers were found at 1 dpi in the co-inoculated groups versus the single *v*NDV inoculated group (*P <* 0.05). However, birds in the simultaneously inoculated group did not shed virus by the CL route at 2 dpi compared to 10 of 10 birds shedding virus by this route in the single virus inoculated group.

The number of birds shedding HPAIV by the OP and CL route, and the titers of virus shed, were similar at both 1 and 2 dpi among groups inoculated only with HPAIV and groups simultaneously co-infected with NDV, with the exception of birds co-inoculated with *l*NDV which shed higher titers of HPAIV by the CL route (*P* < 0.05). Similarly, no difference in the number of birds shedding virus by the OP route was found between birds sequentially inoculated with *l*NDV or *m*NDV and HPAIV compared to HPAIV only inoculated birds.

Strikingly, all birds inoculated with *v*NDV (low and high dose) then sequentially inoculated with HPAIV, showed a reduction in the number of birds shedding HPAIV by the OP and CL routes (*P <* 0.001; *P* < 0.0001), and a reduction of viral titers (*P <* 0.05) when examined at 1 dpi, indicating that HPAIV replication was hampered by *v*NDV. However, this effect was short-lived since no difference between sequentially inoculated and single-virus-inoculated groups was observed as of 2 dpi and beyond.

#### Gross, microscopic lesions and viral antigen staining in tissues

Two birds from each group were necropsied 2 dpi (or 2 days after inoculation with HPAIV in the groups that received the viruses sequentially). No gross lesions were observed at 2 dpi in chickens inoculated with the sham inoculum. Mild sinusitis and conjunctivitis were present in all birds inoculated with *l*NDV or the *m*NDV. Chickens inoculated with the *v*NDV had severe sinusitis, edema and hemorrhages in eyelids, and enlarged mottled spleens. Birds infected with HPAIV, regardless of exposure to NDV, had sinusitis, hemorrhages on serosal surfaces of internal organs, especially in the coronary fat and on the epicardium, within the pectoral muscles and in the cecal tonsils and Meckel’s diverticulum; swollen kidneys; enlarged mottled spleens; and malacic brains. Lesions were slightly more severe in birds simultaneously inoculated with *v*NDV and HPAIV.

Microscopic lesions in chickens inoculated with *l*NDV and *m*NDV were confined to the sites of virus inoculation and included mild catarrhal rhinitis, sinusitis, and mild edema of the eyelid. By contrast, more severe and widespread histopathological findings were seen with *v*NDV and HPAIV infections, consistent with previous descriptions [24,31].

**Figure 2:**
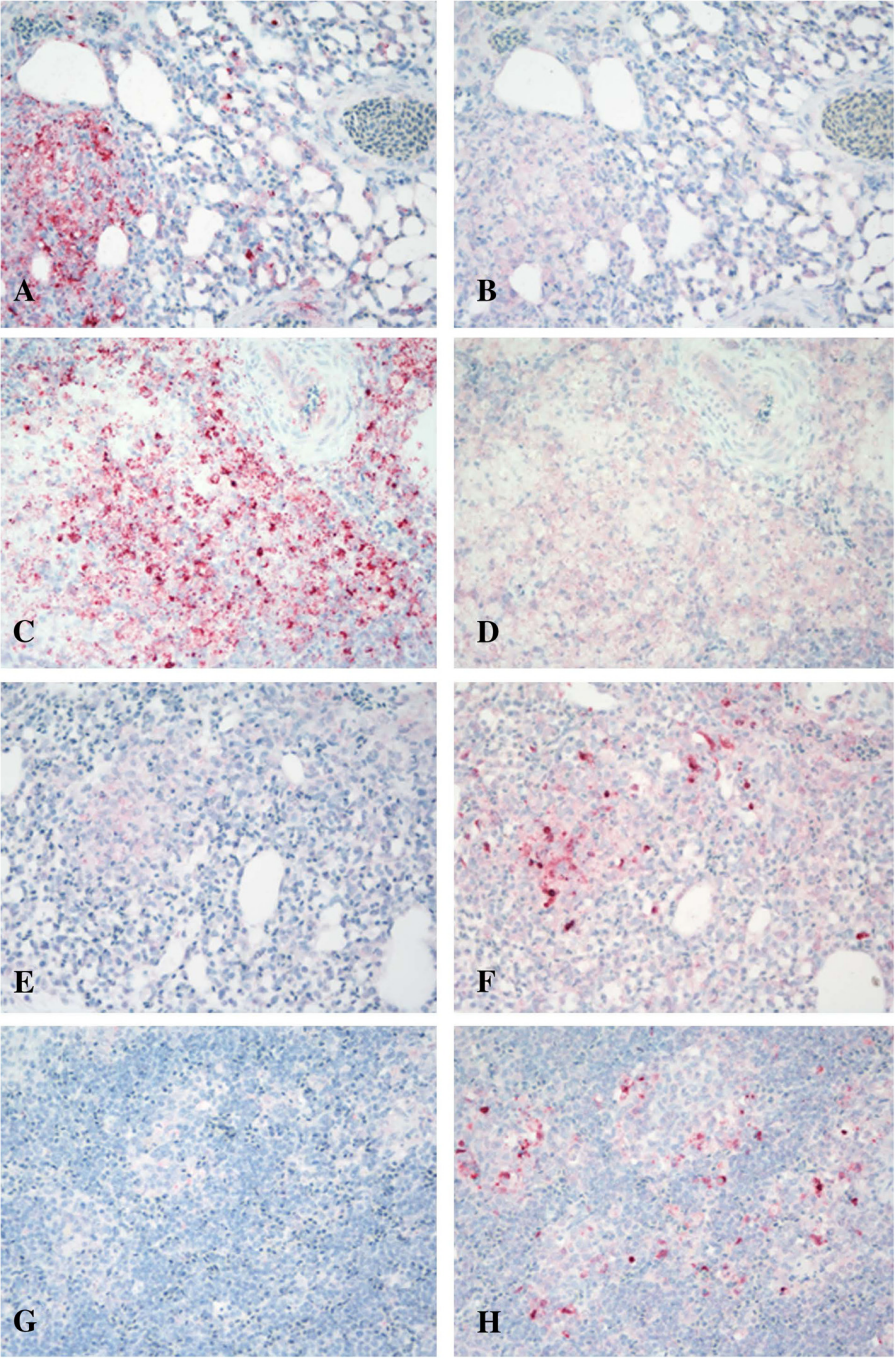
**Immunohistochemical staining for NDV and HPAIV antigens in tissues of co-infected chickens.** Viral antigens are stained red. Spleen and lung from a chicken sequentially inoculated with a low virulence *l*NDV (LaSota strain) and a HPAIV (A/Ck/Queretaro/14588-19/95 H5N2); tissues collected 2 days after HPAIV inoculation (**A** and **B**, lung; **C** and **D** spleen). Strong virus staining for HPAIV (**A** and **C**) but not for NDV (**B** and **D**). HPAIV antigen staining found in epithelium of air capillaries, mononuclear cells and necrotic debris in lungs, and in mononuclear cells and necrotic debris in spleen. Spleen and lung from a chicken sequentially inoculated with a virulent *v*NDV (CA2002 strain) and a HPAIV (A/Ck/Queretaro/14588-19/95 H5N2); tissues collected 2 days after HPAIV inoculation (**E** and **F**, lung; **G** and **H** spleen). Strong antigen staining for NDV (**F** and **H**) but not for HPAIV (**E** and **G**). NDV antigen staining found in mononuclear cells and necrotic debris in lung and in the histiocytes surrounding the penicillary arteries in spleen. Magnifications, 400X.

Viral antigen was present in several organs of birds inoculated with *m*NDV, *v*NDV or HPAIV, suggesting systemic infection with all three viruses (Additional files 1 and 2, Figure 2). In birds inoculated with NDV, NDV-nucleoprotein (NDV-NP) antigen staining was intracytoplasmic. The tissues with the strongest staining were; the nasal cavity, eyelids, and lymphoid organs, similar to previously described [24]. In birds inoculated with the HPAIV, AIV-nucleoprotein (AIV-NP) antigen was detected intranuclear and intracytoplasmic in blood vessel endothelial cells throughout the body, and in various cell types within areas of necrosis and inflammation in many tissues including nasal cavity, lymphoid tissues, lung, brain, liver, and spleen, similar lo previously described [31]. Specifically, virus antigen was present in parenchymal cells of some organs including nasal epithelium, cardiac myocytes, Kupffer cells, hepatocytes, microglial cells and neurons, respiratory epithelial cells in the lung, and tubular epithelial and glomerular cells of the kidney.

Compared to single-virus infected birds, tissues from birds simultaneously infected with *l*NDV and HPAIV showed more widespread AIV-NP antigen staining. Simultaneous co-infection with *m*NDV and HPAIV and low dose *v*NDV and HPAIV showed no differences in HPAIV-NP antigen staining compared with single-virus infected birds; however, in birds simultaneously co-infected with high dose *v*NDV and HPAIV there was widespread NDV-NP and AIV-NP antigen staining in tissues. Regarding birds sequentially infected with the *l*NDV and HPAIV, and taking into account that tissues were examined 2 dpi after HPAIV inoculation (4 dpi for NDV), an enhanced AIV-NP antigen staining was observed when compared to birds infected only with HPAIV. Widespread AIV-NP antigen staining was found both in spleen and lung (Figure 2). In birds simultaneously infected with *m*NDV and HPAIV, AIV-NP antigen staining was less widespread than in birds infected only with HPAIV alone. Interestingly, in birds sequentially infected with *v*NDV (low and high dose) and HPAIV, AIV-NP antigen staining in tissues was minimal and NDV-NP widespread (Figure 2).

#### Virus titers in lung and spleen

Viral titers were determined in lungs and spleens collected from 2 birds per group at 2 dpi (Additional file 3). NDV viral titers were under the limit of detection in tissues from *l*NDV and *m*NDV single-inoculated and simultaneously inoculated birds. Low to moderate NDV virus titers were present in the lungs and spleens of chickens inoculated with *v*NDV given single or co-infected simultaneously with HPAIV (2–4 log_10_ EID_50_/gr); but NDV titers in spleen and lung were under the limit of detection in birds simultaneously infected with the low dose of *v*NDV and HPAIV. NDV titers were higher in the sequentially inoculated groups because it corresponded to 4 dpi after NDV inoculation. At this time, *l*NDV and *m*NDV were detected in tissues. High HPAIV titers were present in both lung and spleen collected from all birds inoculated with HPAIV; however birds previously infected with *v*NDV (low or high dose) had lower HPAIV titers (log_10_ 3.3-4.2 EID_50_/gr) than the other HPAIV-inoculated groups (log_10_ 4.4-8.2 EID_50_/gr).

### Study 2

Based on the slight delay of mortality time observed in the group sequentially infected with *m*NDV and HPAIV, and to accentuate the effects of NDV on HPAIV replication, a study was performed in which 3-week-old chickens were inoculated with the same *m*NDV (Pigeon 1984) followed by the same HPAIV (A/chicken/Queretaro/14588-1988 H5N2) three days later. The one day delay in the HPAIV challenge allows *m*NDV pigeon isolate to achieve more replication as the viral titers typically don’t reach their peak until 4 dpi. The titer of the HPAIV inoculum was also reduced to one log lower (10^5.3^ EID_50_) than in the previous experiment to more likely observe differences between groups. Most birds inoculated with *m*NDV were protected against HPAIV disease, with only 1 of 12 birds dying at 8 dpi in the sequential co-infected group, compared to 11 of 12 birds dying in the group that received the HPAIV alone (MDT of 3.5 days). In addition, while all the chickens in the HPAIV-inoculated group were shedding virus at 2 dpi, only 1 bird out of 12 was positive for HPAIV in the sequentially co-infected group (data not shown). These results suggested that the timing of exposure and the titers of the viruses might affect the outcome of infection.

### Study 3

A third experiment was performed to determine if the reduction in severity of HPAIV infection in birds previously infected with *m*NDV occurs independently of age and virus strain. Different age chickens (2 and 4-week-old) were used to compare the effect of immune system maturity on the results of the co-infections. Furthermore a different HPAIV isolate (A/chicken/Jalisco/CPA-12283-12/2012 H7N3) was used to determine if the protective effect of *m*NDV applies to other HPAIV strains. Similar to Study 2, a lower dose of HPAIV was used, and the inoculation of HPAIV was performed 3 days after inoculation with the *m*NDV.

#### Clinical signs

**Figure 3:**
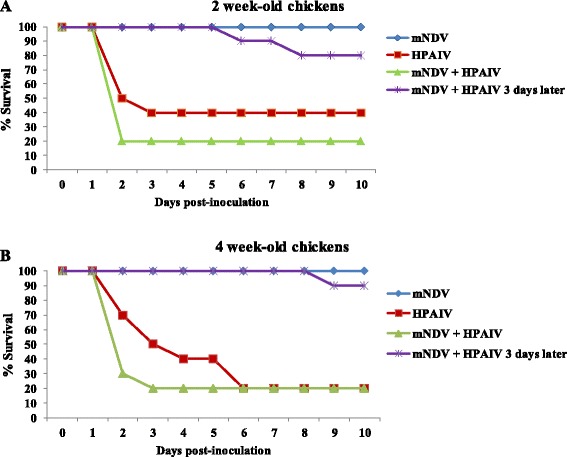
**Survival curves after inoculation of chickens with** ***m*** **NDV and HPAIV (Study 3).** **A**. 2-week-old chickens. **B**. 4-week-old chickens. Chickens inoculated simultaneously or sequentially with *m*NDV and with HPAIV.

None of the birds inoculated with sham inoculum showed clinical signs. Chickens inoculated with the *m*NDV had mild conjunctivitis. Six to 8 out of 10 birds (2 and 4 week-old birds respectively) inoculated only with HPAIV died, with a MDT of 1.6 days for the 2-week-old chickens and 3.5 days for the 4-week-old birds (Tables 2 and 3, Figure 3). Eight of 10 birds died in the groups that were inoculated simultaneously with the *m*NDV and the HPAIV, with MDT’s of 2 or 2.1 days. In general, chickens inoculated with the HPAIV virus in these groups in both experiments had similar clinical signs, including ruffled feathers, lethargy, anorexia and prostration and some birds presented respiratory distress, swollen head, and cyanotic comb, wattles and legs. Most birds died without showing previous clinical signs (peracute disease). Only 1 or 2 of 10 birds 2 and 4-week-old birds respectively) died in the groups sequentially inoculated with the viruses, with a MDT of 7 days for the 2-week-old birds and 9 for the 4-week-old birds. The surviving birds in these groups showed no clinical signs.

**Table 2:** **Study 3: Mortality, mean death time (MDT), and OP virus shedding in 2 and 4 week-old chickens inoculated with** ***m*** **NDV and HPAIV**

**Age**	**Virus**	**Number of dead/total (MDT)**	**OP shedding: number of birds shedding/total (Log**_**10**_ **EID**_**50**_ **/mL)** ^**a**^							
			**NDV**				**HPAIV**			
			**2 dpi**	**3 dpi**	**4 dpi**	**7 dpi**	**2 dpi**	**3 dpi**	**4 dpi**	**7 dpi**
2 weeks old	HPAIV	6/10 (1.6)	nd	nd	nd	nd	5/7 (7.9 ± 3)^A^	3/4 (1.9 ± 0.4)^A^	0/4^A^	0/4^A^
	*m*NDV	-	9/10 (3.7 ± 0.3)^A^	9/10 (3.7 ± 0.1)^A^	10/10 (4.3 ± 0.3)^A^	10/10 (4.8 ± 0.2)^A^	nd	nd	nd	nd
	*m*NDV + HPAIV	8/10 (2)	4/4 (3.3 ± 0.6)^A^	2/2 (4.1 ± 0.1)^A^	2/2 (4.5 ± 0.4)^A^	2/2 (3.8 ± 0.2)^A^	3/4 (7.3 ± 2.9)^A^	0/2^A^	0/2^A^	0/2^A^
	*m*NDV + HPAIV 3 days later	2/10 (7.0)	10/10 (3.4 ± 0.3)^A^	9/10 (3.6 ± 0.3)^A^	9/9 (3.6 ± 0.3)^A^	9/9 (4.8 ± 0.2)^A^	0/10**^B^	0/10*^A^	1/9 (4.5)^A^	1/9 (9.3)^A^
4 weeks old	HPAIV	8/10 (3.5)	nd	nd	nd	nd	8/9 (7.1 ± 0.9)^A^	0/5^A^	4/4 (3.3 ± 0.8)^A^	0/2^A^
	*m*NDV	-	10/10 (3.5 ± 0.2)^A^	8/8 (3.5 ± 0.2)^A^	8/8 (3.8 ± 0.3)^A^	8/8 (4.7 ± 0.2)^A^	nd	nd	nd	nd
	*m*NDV + HPAIV	8/10 (2.1)	3/5 (2.7 ± 0.5)^A^	2/2 (3 ± 0.9)^A^	2/2 (2.9 ± 0.7)^A^	2/2 (3.6 ± 0.8)^A^	3/5 (6.6 ± 1.7)^A^	0/2^A^	0/2^A^	0/2^A^
	*m*NDV + HPAIV 3 days later	1/10 (9.0)	9/10 (3.9 ± 0.3)^A^	8/10 (3.4 ± 0.4)^A^	9/10 (3.5 ± 0.3)^A^	10/10 (4.5 ± 2.1)^A^	0/10***^B^	0/10 ^A^	0/10***^A^	1/10 (10)^A^

For groups inoculated sequentially with the viruses, the HPAIV results are 2, 3, 4, and 7 days after H7N3 HPAIV inoculation which corresponds to days 5, 6, 7 and 10 after NDV inoculations.

^a^Viral titers average ± SEM from the positive birds; Log_10_ EID_50_ equivalents were determined by qRRT-PCR.

nd = not done.

*Significant difference in number of chickens virus positive compared to single virus infected groups (*P < 0.05; **P < 0.01; ***P < 0.001).

^A; B^Different superscript uppercase denote significant difference in virus titers compared to single virus infected groups (*P* < 0.05).

**Table 3:** **Study 3: CL virus shedding in 2 and 4 week-old chickens inoculated with** ***m*** **NDV and HPAIV**

**Age**	**Virus**	**CL shedding: number of birds shedding/total (Log**_**10**_ **EID**_**50**_ **/mL)** ^**a**^							
		**NDV**				**HPAIV**			
		**2 dpi**	**3 dpi**	**4 dpi**	**7 dpi**	**2 dpi**	**3 dpi**	**4 dpi**	**7 dpi**
2 weeks old	HPAIV	nd	nd	nd	nd	3/7 (9.8 ± 0.6)^A^	0/4^A^	0/4^A^	0/4^A^
	*m*NDV	0/10^A^	8/10 (2.8 ± 0.3)^A^	8/8 (3.6 ± 0.2)^A^	10/10 (3.6 ± 0.2)^A^	nd	nd	nd	nd
	*m*NDV + HPAIV	0/4^A^	2/2 (2.1 ± 0.4)^A^	2/2 (3.7 ± 0.2)^A^	2/2 (4.5 ± 0.15)^A^	2/4 (8.5 ± 2.8)^A^	0/2^A^	0/2^A^	0/2^A^
	*m*NDV + HPAIV 3 days later	1/9 (2.1)^A^	8/10 (2.9 ± 0.1)^A^	10/10 (3.9 ± 0.2)^A^	9/9 (4.3 ± 0.2)^A^	0/10^B^	0/10^A^	3/9 (2.2 ± 0.5)^A^	1/9 (6.9)^A^
4 weeks old	HPAIV	nd	nd	nd	nd	4/9 (8.1 ± 0.8)^A^	1/5 (5.6)^A^	1/4 (5.4)^A^	0/2^A^
	*m*NDV	0/10	4/8 (1.9 ± 0.1)^A^	8/8 (2.9 ± 0.1)^A^	8/8 (3.9 ± 0.1)^A^	nd	nd	nd	nd
	*m*NDV + HPAIV	1/5 (1.8)	1/2 (2.5)^A^	1/2 (3.2)^A^	2/2 (3.7 ± 0.3)^A^	3/5 (8.1 ± 2.5)^A^	0/2^A^	0/2^A^	0/2^A^
	*m*NDV + HPAIV 3 days later	0/10	5/10 (2.3 ± 0.2)^A^	10/10 (3.7 ± 0.2)*^B^	10/10 (4.2 ± 0.2)^A^	0/10*^B^	1/10 (1.6)^A^	0/10^A^	1/10 (4)^A^

For groups inoculated sequentially with the viruses, the HPAIV results are 2, 3, 4, and 7 days after HPAIV inoculation which corresponds to days 5, 6, 7 and 10 after NDV inoculations.

^a^Viral titers average ± SEM from the positive birds; Log_10_ EID_50_ equivalents were determined by qRRT-PCR.

nd = not done.

*Significant difference in number of chickens virus positive compared to single virus infected groups (* *P* < 0.05).

^A; B^Different superscript uppercase denote significant difference in virus titers compared to single virus infected groups (*P* < 0.05).

No significant difference in survival among groups was found regarding the age of the birds and both behaved similarly. None of the 2-week and 4-week-old chickens inoculated only with *m*NDV died; thus, they showed statistically significant differences in survival when compared to the HPAIV-single-infected birds (2-week-old, *P* < 0.05; 4-week old *P* < 0.001), and those simultaneously co-infected with HPAIV (2 and 4-week-old, *P* < 0.001). When HPAIV was given 3 days after the *m*NDV, almost all birds survived and the birds that died had a longer mean death time when compared to the group that was only inoculated with the HPAIV (2 and 4-week-old; *P* < 0.001).

#### Viral shedding

The number of birds shedding virus by the OP and CL routes and virus titers at 2, 3, 4, and 7 dpi are shown in Tables 2 and 3. Birds sequentially co-infected with *m*NDV and HPAIV were evaluated at days 5, 6, 7 and 10 of the experiment, corresponding to days 2, 3, 4, and 7 after HPAIV inoculation. All 2-week-old chickens inoculated with *m*NDV and co-infected or not with HPAIV, were shedding NDV by the OP route at the time points examined, and most CL swabs were positive by 3 dpi. No significant difference in *m*NDV titers was found in OP and CL swabs when comparing groups. Some of the 4-week-old birds co-infected with *m*NDV and HPAIV were not shedding *m*NDV but the number of birds shedding was not different than *m*NDV only-inoculated birds, with the exception of the group sequentially inoculated with the viruses, in which more birds shed virus by the CL route at 4 dpi and at higher titers. This difference can be attributed to the fact that this time point corresponds to day 7 after *m*NDV inoculation.

At 2 dpi, both 2 and 4-week-old chickens in the groups sequentially infected with *m*NDV and HPAIV lacked HPAIV OP and CL shedding, significantly lower than to the groups that only received HPAIV (*P* < 0.001, *P* < 0.0001). Furthermore, at 3, 4 and 7 dpi, only 0 to 3 out of 9 or 10 birds from these groups (2 and 4-week-old birds respectively) were shedding HPAIV by the OP or CL route. In the groups simultaneously infected with *m*NDV and HPAIV, there was no significant reduction in the number of surviving birds shedding HPAIV or the viral titers shed at 2 dpi compared to the groups that only received HPAIV. However, 4 of 4 4-week-old birds inoculated only with the HPAIV were still shedding by the OP route at 4 dpi while none of the co-infected birds were.

#### Gross, microscopic lesions and viral antigen staining in tissues

Two birds from each group were necropsied at day 2 or 2 days after inoculated with the HPAIV in the groups that got the viruses sequentially. No gross lesions were observed in chickens inoculated with the sham inoculum, and mild conjunctivitis and sinusitis was present in the birds inoculated with *m*NDV or sequentially co-infected with *m*NDV and HPAIV. Chickens inoculated only with HPAIV or co-infected with *m*NDV and HPAIV simultaneously, presented lesions typical of HPAIV infection as described in Study 1.

Histopathological findings were consistent with *m*NDV and HPAIV infections [3,25]. No or less severe lesions were observed in the birds sequentially inoculated with the viruses compared to birds that received only HPAIV or were inoculated simultaneously with the viruses. Mild conjunctivitis and sinusitis was present in birds inoculated only with *m*NDV. Mild to severe, diffuse catarrhal rhinitis and mild to moderate tracheitis was present in all birds inoculated only with HPAIV or simultaneously inoculated with *m*NDV. The rest of the lesions were similar to those observed in study 1.

By immunohistochemistry, NDV-NP antigen was detected mostly in the nasal cavity, lung, spleen and cecal tonsils, but also in liver and intestine in birds inoculated only with *m*NDV. NDV-NP antigen staining was more common in tissues from birds co-infected with the HPAIV, including viral staining in eyelid and trachea (Additional file 4). Widespread AIV-NP antigen staining was present in tissues from birds inoculated with the HPAIV given alone or given simultaneously with *m*NDV (Additional file 5). In contrast, AIV-NP staining was limited to nasal cavity, lung and spleen in birds receiving the viruses sequentially.

#### Virus titers in lung and spleen

Viral titers were determined in lungs and spleens collected from the 2 birds per group necropsied at 2 dpi (Additional file 6). Similar to study 1, HPAIV titers were generally lower in both lung and spleen in birds sequentially inoculated with *m*NDV and HPAIV (3.3-5.2 log_10_ EID_50_/gr) when compared to groups only receiving HPAIV (6.2-8.1 log_10_ EID_50_/gr).

#### Serology

HI assays were used to test for antibodies against AIV and NDV in surviving birds in study 3. Because serum samples were not taken the same day for single-virus, simultaneously and sequentially exposed birds (10 dpi for the first two and 7 dpi for the second), the level of the HI titers couldn’t be strictly compared, but antibody titers provided an alternative method for determining viral infection. All the surviving chickens seroconverted to *m*NDV, but not to HPAIV when exposed, with no differences in titers among the treatment groups (data not shown).

## Discussion

The goal of these studies was to evaluate the effect of NDV on HPAIV infection in chickens. Our results showed that the severity of the disease caused by HPAIV, the number of birds shedding virus and the titers of virus shed can be reduced by previous infection with virulent strains of NDV (*m*NDV and *v*NDV) if given 2–3 days before HPAIV challenge.

As expected, single-virus infection with *l*NDV and *m*NDV did not cause disease or death in chickens. The *m*NDV isolate used in this study was a pigeon strain (PPMV-1), which typically cause high mortality in pigeons but not in chickens. These viruses can acquire virulence after multiple passages in chickens [32]. In contrast, infection with *v*NDV or HPAIV caused severe disease and high mortality (60-100%). In study 1, there were no differences in mortality rates in groups that received HPAIV alone and sequentially and simultaneously infected groups, but increased MDT’s were observed in groups co-infected with *l*NDV, *m*NDV and low dose of *v*NDV. In addition, OP and CL HPAIV shedding at 1 dpi were significantly lower in the sequential *v*NDV (low or high dose) and HPAIV-inoculated groups when compared to the group inoculated only with HPAIV or simultaneously with *v*NDV and HPAIV. By 2 dpi, the OP and CL viral shedding were similar in all groups, but IHC on tissues from sequentially infected groups showed little or no HPAIV antigen as compared to high levels of HPAIV antigen in the other groups. This indicates that although *v*NDV interfered with initial HPAIV replication, the HPAIV challenge was sufficiently high to overcome *v*NDV interference and killed the chickens, the *v*NDV itself killed the chickens, or a combination of both. In studies 2 and 3, we found that when HPAIV was given at a lower dose three days after inoculation with the less virulent NDV (*m*NDV), bird survival increased and virus shedding decreased. By using this less virulent NDV strain that also replicates systemically, the replication of HPAIV was reduced to a point that allowed the host immune response to control the infection and prevent death.

These results agree with our previous studies showing viral interference between less pathogenic forms of NDV and AIV in chickens and turkeys [21]. In this previous study, although no differences in clinical signs were observed in chickens, prior exposure to *l*NDV modified viral shedding patterns by delaying the LPAIV shedding. In the present study, *l*NDV had little effect on HPAIV replication, failing to stop viral systemic spread and replication leading to death. Nevertheless, the presence of HPAIV affected *l*NDV replication when given simultaneously.

In studies 2 and 3, *m*NDV given 3 days prior to a lower dose HPAIV challenge interfered with HPAIV replication protecting chickens against death. *m*NDV and *v*NDV strains have a multi-basic amino acid motif at the fusion cleavage site and can be cleaved intracellularly by ubiquitous furin-like proteases found in most host tissues [33]. This results in a systemic infection that often is fatal with *v*NDV’s. On the other hand, *m*NDV’s are less pathogenic, rarely producing neurological disease, and death only seen in young birds [3]. Both NDV and AIV have mechanisms to interfere with the host innate immune responses including reduction of the interferon (IFN) response. The V protein of NDV is an IFN antagonist [34], but this effect appears to be strain dependent. For instance, the V protein of the mesogenic Beaudette C strain exhibits a greater antagonistic effect on IFN induction in vitro than that of the lentogenic LaSota strain [35]. Interestingly, in the case of the velogenic strain CA02, a block of IFN pathways did not occur at the mRNA level when the chicken immune response was evaluated by microarray, in fact CA02 elicited a strong immune response in chickens suggesting that the host response itself may contribute to the pathogenesis of *v*NDV [36]. The most likely mechanism for the viral interference observed is that the *m*NDV produced a robust IFN response that resulted in blocking or greatly reducing HPAIV viral replication in the sequentially infected birds. In the simultaneously infected birds, the IFN response did not develop in time to prevent infection, resulting in death of the birds. In the present study, *m*NDV might be eliciting a more balanced innate immune response, not as strong as *v*NDV but strong enough to activate the IFN pathway and protect against a second virus infection. In this scenario, although it is known that HPAIV can delay IFN-induced antiviral responses [37], the overall outcome of HPAIV infection depends on the early innate immune protection induced by the *m*NDV, therefore, the timing of the previous infection with *m*NDV might be crucial for protection against HPAIV infection. Because of strain differences, the observations presented cannot be generalized to all *m*NDV’s and the interfering potential of other *m*NDV strains needs to be evaluated.

Age-related susceptibility to disease in birds may be associated with a maturing immune system. For instance, the expression levels of components involved in the IFN system appear to increase with age [38]. However, in this study, 2 and 4-week-old chickens showed similar survival rates and virus shedding patterns suggesting no difference in immune competency. These results, although insufficient to completely characterize the mechanism involved in suppression of viral replication, might also suggest that, apart from non-specific innate immune responses induced by the earlier infection, viral competition for target host cells could also be involved, this second alternative also needing further study.

The existence of previous viral-host interactions capable of modifying the outcomes of a sequential HPAIV infection indicates that it may be possible to develop novel strategies to prevent or reduce mortality in HPAIV-infected naïve birds. In some countries where ND is endemic, *m*NDV live vaccine strains are used to control the disease in poultry [3,39]. Some of these countries report outbreaks of HPAIV; therefore it’s possible that co-infections with *m*NDV occur. If this was the case, disease and mortality caused by HPAIV could be curbed by previous infection with *m*NDV, affecting the clinical diagnosis and the control of the virus; but, as mentioned, the effect of *m*NDV on HPAIV infection is most likely strain dependent. Vaccination with *m*NDV strains to protect against HPAI could be a possible option in countries where ND is endemic. However, since virulent strains of NDV are reportable to the OIE, the use of *m*NDV vaccines would not be an option in countries free of ND. Further studies are needed to explore this unconventional use of live NDV vaccines.

In conclusion, co-infection with NDV and HPAIV can affect the viral replication dynamics and the disease caused by these viruses in chickens, but this effect will depend on the virulence of the viruses involved, the challenge titer of the viruses and the timing of the co-infections. The identification of factors that influence a delay of infection of one virus by another will provide new insights in the pathogenesis of these viruses, allowing the development of novel ways to control viruses.

## Additional files

Additional file 1:
**Study 1: average distribution of NDV-NP antigen by IHC.** Tissues from chickens inoculated simultaneously or sequentially with different strains of NDV and with a HPAIV were examined at 2 dpi in single and simultaneously infected groups and at 2 days after inoculation with the HPAIV in groups sequentially infected (bird 1/bird 2). Additional file 2:
**Study 1: average distribution of AIV-NP antigen by IHC.** Tissues from chickens inoculated simultaneously or sequentially with different strains of NDV and with a HPAIV were examined at 2 dpi in single and simultaneously infected groups and at 2 days after inoculation with the HPAIV in groups sequentially infected (bird 1/bird 2). Additional file 3:
**Study 1: comparison of virus titers in lung and spleen.** Tissues taken from 2 birds per group at 2 dpi. For groups inoculated sequentially with the viruses, HPAIV tissues are 2 days after HPAIV inoculation which corresponds to 4 days after NDV inoculation (bird 1/bird 2). Additional file 4:
**Study 3: average distribution of NDV-NP antigen by IHC.** Tissues from chickens inoculated simultaneously or sequentially with *m*NDV and with a HPAIV. Single and simultaneously infected groups were analyzed at 2 dpi and at 2 days after inoculation with the HPAIV in groups sequentially infected (bird 1/bird 2). Additional file 5:
**Study 3: average distribution of AIV-NP antigen by IHC in tissues.** Tissues from chickens inoculated simultaneously or sequentially with mNDV and with a HPAIV were examined. Single and simultaneously infected groups were analyzed at 2 dpi and at 2 days after inoculation with the HPAIV in groups sequentially infected (bird 1/bird 2). Additional file 6:
**Study 3: comparison of virus titers in lung and spleen.** Tissues taken from 2 birds per group at 2 dpi. For groups inoculated sequentially with the viruses, HPAIV results are 2 days after HPAIV inoculation (bird 1/bird 2).
