# Supplementary material for: Previous infection with virulent strains of Newcastle disease virus reduces highly pathogenic avian influenza virus replication, disease, and mortality in chickens
Source: Vet Res. 2015 Sep 23;46(1):97. doi: 10.1186/s13567-015-0237-5 (PMC4579609; doi:10.1186/s13567-015-0237-5)
Supplement: Additional file 1: — Study 1: average distribution of NDV-NP antigen by IHC. Tissues from chickens inoculated simultaneously or sequentially with different strains of NDV and with a HPAIV were examined at 2 dpi in single and simultaneously infected groups and at 2 days after inoculation with the HPAIV in groups sequentially infected (bird 1/bird 2). [file 13567_2015_237_MOESM1_ESM.docx]

| Virus | Detection of NDV antigen in tissues | | | | | | | | | | | |
| --- | --- | --- | --- | --- | --- | --- | --- | --- | --- | --- | --- | --- |
|  | Nasal  cavity | Eyelid | Trachea | Lung | Heart | Spleen | Cecal tonsils | Liver | Intestine | Bursa | Kidney | Brain |
| *l*NDV | +/+ | ++/+ | -/- | -/- | -/- | -/- | +/- | -/- | +/- | -/- | -/- | -/- |
| *m*NDV | ++/++ | +/+ | +/+ | -/- | -/- | -/- | ++/+ | +/- | +/+ | -/- | -/- | -/- |
| *v*NDV low dose | ++/++ | +/++ | +/+ | +/+ | -/- | +/+ | +++/+ | +/+ | +/+ | -/- | -/- | -/- |
| *v*NDV high dose | ++/++ | +++/++ | +/+ | +/++ | -/- | +/+ | +++/++ | ++/++ | +/++ | +/+ | -/- | +/+ |
| HPAIV | nd | nd | nd | nd | nd | nd | nd | nd | nd | nd | nd | nd |
| *l*NDV + HPAIV | +/- | ++/+ | -/+ | -/- | -/- | -/- | +++/++ | +/- | +/+ | +/- | -/- | -/- |
| *m*NDV + HPAIV | +/+ | ++/+ | +/+ | -/- | -/- | -/- | +/+ | +/- | +/+ | -/- | -/- | -/- |
| *v*NDV low dose + HPAIV | +/+ | ++/++ | +/+ | -/- | -/- | -/- | +/++ | +/+ | +/+ | +/+ | -/- | -/- |
| *v*NDV high dose + HPAIV | +++/++ | +++/++ | +/+ | +/+ | +/- | +/++ | ++/+++ | +/+ | +/+ | ++/+ | -/- | -/- |
| *l*NDV + HPAIV 2 days later | ++/+ | ++/+ | +/+ | ++/++ | -/- | ++/- | ++/+ | +/+ | +/+ | +/+ | -/- | -/- |
| *m*NDV + HPAIV 2 days later | ++/+ | ++/+ | +/+ | ++/- | -/- | +/+ | +/++ | -/- | +/+ | -/- | -/- | -/- |
| *v*NDV low dose + HPAIV 2 days later | +++/+++ | +++/++ | +++/++ | +++/++ | -/- | ++/++ | +++/++ | +/+ | +++/++ | +++/++ | +/- | +/++ |
| *v*NDV high dose + HPAIV 2 days later | +++/+++ | +++/++ | +++/++ | +++/+++ | +/- | ++/++ | +++/++ | +/+ | +++/++ | +++/++ | +/- | ++/++ |

nd = not done. −

=

no positive cells; +

=

single positive cells; ++

=

scattered groups of positive cells; +++

=

widespread positivity.
