# Supplementary material for: Previous infection with virulent strains of Newcastle disease virus reduces highly pathogenic avian influenza virus replication, disease, and mortality in chickens
Source: Vet Res. 2015 Sep 23;46(1):97. doi: 10.1186/s13567-015-0237-5 (PMC4579609; doi:10.1186/s13567-015-0237-5)
Supplement: Additional file 2: — Study 1: average distribution of AIV-NP antigen by IHC. Tissues from chickens inoculated simultaneously or sequentially with different strains of NDV and with a HPAIV were examined at 2 dpi in single and simultaneously infected groups and at 2 days after inoculation with the HPAIV in groups sequentially infected (bird 1/bird 2). [file 13567_2015_237_MOESM2_ESM.docx]

| Virus | Detection of AIV antigen in tissues | | | | | | | | | | | |
| --- | --- | --- | --- | --- | --- | --- | --- | --- | --- | --- | --- | --- |
|  | Nasal cavity | Eyelid | Trachea | Lung | Heart | Spleen | Cecal tonsils | Liver | Intestine | Bursa | Kidney | Brain |
| *l*NDV | nd | nd | nd | nd | nd | nd | nd | nd | nd | nd | nd | nd |
| *m*NDV | nd | nd | nd | nd | nd | nd | nd | nd | nd | nd | nd | nd |
| *v*NDV low dose | nd | nd | nd | nd | nd | nd | nd | nd | nd | nd | nd | nd |
| *v*NDV high dose | nd | nd | nd | nd | nd | nd | nd | nd | nd | nd | nd | nd |
| HPAIV | +++/++ | ++/++ | +/+ | +++/+++ | +/+ | ++/++ | +/+ | +/- | +/- | +/+ | -/+ | ++/++ |
| *l*NDV + HPAI | ++/++ | ++/++ | ++/+ | +++/+++ | +++/++ | +++/+++ | +++/++ | ++/++ | ++/+ | +/+ | -/- | ++/++ |
| *m*NDV + HPAIV | ++/++ | ++/++ | +/+ | +++/+++ | ++/+ | ++/++ | ++/+ | +/+ | +/+ | +/- | -/- | +++/++ |
| *v*NDV low dose + HPAIV | +/++ | ++/++ | +/- | +++/+++ | -/+ | ++/+ | +/++ | +/- | -/- | +/- | -/- | +/++ |
| *v*NDV high dose + HPAIV | ++/++ | +++/++ | +/+ | +++/+++ | +/+ | +++/+++ | +++/++ | ++/+ | +/++ | ++/+ | +/+ | +++/++ |
| *l*NDV + HPAIV 2 days later | ++/++ | +++/+ | ++/+ | +++/+++ | +/+ | +++/++ | +++/++ | ++/++ | +/+ | +/+ | +/+ | +++/++ |
| *m*NDV + HPAIV 2 days later | ++/+ | ++/+ | +/+ | ++/++ | +/- | +/++ | +/- | -/- | -/- | -/- | -/- | ++/- |
| *v*NDV low dose + HPAIV 2 days later | +/++ | -/- | +/+ | +/+ | -/- | -/- | -/- | -/- | -/- | -/- | -/- | -/- |
| *v*NDV high dose + HPAIV 2 days later | +/+ | -/- | +/+ | +/- | -/- | -/- | -/- | -/- | -/- | -/- | -/- | -/- |

nd = not done. −

=

no positive cells; +

=

single positive cells; ++

=

scattered groups of positive cells; +++

=

widespread positivity.
