# Supplementary material for: Previous infection with virulent strains of Newcastle disease virus reduces highly pathogenic avian influenza virus replication, disease, and mortality in chickens
Source: Vet Res. 2015 Sep 23;46(1):97. doi: 10.1186/s13567-015-0237-5 (PMC4579609; doi:10.1186/s13567-015-0237-5)
Supplement: Additional file 3: — Study 1: comparison of virus titers in lung and spleen. Tissues taken from 2 birds per group at 2 dpi. For groups inoculated sequentially with the viruses, HPAIV tissues are 2 days after HPAIV inoculation which corresponds to 4 days after NDV inoculation (bird 1/bird 2). [file 13567_2015_237_MOESM3_ESM.docx]

| Virus | Virus titers (Log 10 EID_50_/g) | | | |
| --- | --- | --- | --- | --- |
|  | Lung | | Spleen | |
|  | NDV | HPAIV | NDV | HPAIV |
| *l*NDV | -^a^/- | nd^b^ | -/- | nd |
| *m*NDV | -/- | nd | -/- | nd |
| *v*NDV low dose | 2.5/2.4 | nd | 4.2/4.3 | nd |
| *v*NDV high dose | 2.7/3.7 | nd | 4.2/4.2 | nd |
| HPAIV | nd | 5.9/6.4 | nd | 5/5.3 |
| *l*NDV + HPAI | -/- | 7.7/8.0 | -/- | 7.6/8.1 |
| *m*NDV + HPAIV | -/- | 7.3/5.9 | -/- | 5.5/6.3 |
| *v*NDV low dose + HPAIV | -/- | 7.1/7.3 | -/- | 6.1/2.5 |
| *v*NDV high dose + HPAIV | 2.8/2.9 | 7.8/8.2 | 4.0/5.2 | 7.5/7.6 |
| *l*NDV + HPAIV 2 days later | 3.6/3.6 | 7.6/7.3 | 4.9/1.6 | 6.0/5.7 |
| *m*NDV + HPAIV 2 days later | 5.0/5.3 | 5.6/6.8 | 3.6/4.6 | 4.4/5.7 |
| *v*NDV low dose + HPAIV 2 days later | 5.9/5.5 | 4.2/3.7 | 3.7/3.8 | 3.4/3.3 |
| *v*NDV high dose + HPAIV 2 days later | 6.7/6.2 | 4.2/2.5 | 3.5/3.6 | 4.0/3.6 |

^a^ - = negative; ^b^ nd= not done
