# Supplementary material for: Previous infection with virulent strains of Newcastle disease virus reduces highly pathogenic avian influenza virus replication, disease, and mortality in chickens
Source: Vet Res. 2015 Sep 23;46(1):97. doi: 10.1186/s13567-015-0237-5 (PMC4579609; doi:10.1186/s13567-015-0237-5)
Supplement: Additional file 4: — Study 3: average distribution of NDV-NP antigen by IHC. Tissues from chickens inoculated simultaneously or sequentially with mNDV and with a HPAIV. Single and simultaneously infected groups were analyzed at 2 dpi and at 2 days after inoculation with the HPAIV in groups sequentially infected (bird 1/bird 2). [file 13567_2015_237_MOESM4_ESM.docx]

| Age | Virus | Detection of NDV antigen in tissues | | | | | | | | | | | |
| --- | --- | --- | --- | --- | --- | --- | --- | --- | --- | --- | --- | --- | --- |
|  |  | Nasal  cavity | Eyelid | Trachea | Lung | Heart | Spleen | Cecal tonsils | Liver | Intestine | Bursa | Kidney | Brain |
| 2 weeks old | HPAIV | nd | nd | nd | nd | nd | nd | nd | nd | nd | nd | nd | nd |
|  | *m*NDV | +/+ | -/- | -/- | -/- | -/- | +/- | +/+ | +/- | +/+ | -/- | -/- | -/- |
|  | *m*NDV + HPAIV | +++/+ | +/+ | ++/+ | -/- | -/- | -/- | ++/+ | +/+ | +/+ | -/- | -/- | -/- |
|  | *m*NDV + HPAIV 3 days later | +++/+++ | +/+ | +/+ | +/+ | +/+ | +/+ | +++/++ | -/- | +/+ | -/- | -/- | -/- |
| 4 weeks old | HPAIV | nd | nd | nd | nd | nd | nd | nd | nd | nd | nd | nd | nd |
|  | *m*NDV | +/+ | -/- | -/- | -/- | -/- | +/- | +/+ | +/- | +/- | -/- | -/- | -/- |
|  | *m*NDV + HPAIV | ++/+++ | +/+ | +/+ | -/- | -/- | ++/++ | +++/++ | -/- | +/+ | -/- | -/- | -/- |
|  | *m*NDV + HPAIV 3 days later | +++/++ | +/+ | -/- | +++/+++ | -/- | +++/++ | +++/++ | -/- | +/+ | -/- | -/- | -/- |

nd = not done. −

=

no positive cells; +

=

single positive cells; ++

=

scattered groups of positive cells; +++

=

widespread positivity.
