# Supplementary material for: Previous infection with virulent strains of Newcastle disease virus reduces highly pathogenic avian influenza virus replication, disease, and mortality in chickens
Source: Vet Res. 2015 Sep 23;46(1):97. doi: 10.1186/s13567-015-0237-5 (PMC4579609; doi:10.1186/s13567-015-0237-5)
Supplement: Additional file 5: — Study 3: average distribution of AIV-NP antigen by IHC in tissues. Tissues from chickens inoculated simultaneously or sequentially with mNDV and with a HPAIV were examined. Single and simultaneously infected groups were analyzed at 2 dpi and at 2 days after inoculation with the HPAIV in groups sequentially infected (bird 1/bird 2). [file 13567_2015_237_MOESM5_ESM.docx]

| Age | Virus | Detection of AIV antigen in tissues | | | | | | | | | | | |
| --- | --- | --- | --- | --- | --- | --- | --- | --- | --- | --- | --- | --- | --- |
|  |  | Nasal  cavity | Eyelid | Trachea | Lung | Heart | Spleen | Cecal tonsils | Liver | Intestine | Bursa | Kidney | Brain |
| 2 weeks old | HPAIV | +++/+++ | +++/++ | ++/+ | +++/+++ | ++/+++ | +++/+ | ++/+++ | +/++ | ++/++ | +++/+ | +/+ | +/+++ |
|  | *m*NDV | nd | nd | nd | nd | nd | nd | nd | nd | nd | nd | nd | nd |
|  | *m*NDV + HPAIV | +++/+ | +++/++ | ++/+ | +++/+++ | ++/++ | +++/+++ | +++/++ | +++/+ | +/+ | +++/+ | ++/+ | ++/+ |
|  | *m*NDV + HPAIV 3 days later | -/- | -/- | -/- | ++/++ | -/- | +/+ | -/- | -/- | -/- | -/- | -/- | -/- |
| 4 weeks old | HPAIV | +++/++ | +++/++ | +/++ | +++/+++ | +++/+++ | +++/+++ | +++/++ | +++/++ | +++/++ | +++/++ | +/+ | ++/++ |
|  | *m*NDV | nd | nd | nd | nd | nd | nd | nd | nd | nd | nd | nd | nd |
|  | *m*NDV + HPAIV | ++/++ | +++/++ | +/+ | +++/++ | ++/+ | ++/+ | +++/++ | ++/+ | +/+ | +++/++ | ++/+ | ++ |
|  | *m*NDV + HPAIV 3 days later | +/- | -/- | -/- | ++/++ | -/- | +/+++ | -/- | -/- | -/- | -/- | -/- | -/- |

nd = not done. −

=

no positive cells; +

=

single positive cells; ++

=

scattered groups of positive cells; +++

=

widespread positivity
