# Supplementary material for: Previous infection with virulent strains of Newcastle disease virus reduces highly pathogenic avian influenza virus replication, disease, and mortality in chickens
Source: Vet Res. 2015 Sep 23;46(1):97. doi: 10.1186/s13567-015-0237-5 (PMC4579609; doi:10.1186/s13567-015-0237-5)
Supplement: Additional file 6: — Study 3: comparison of virus titers in lung and spleen. Tissues taken from 2 birds per group at 2 dpi. For groups inoculated sequentially with the viruses, HPAIV results are 2 days after HPAIV inoculation (bird 1/bird 2). [file 13567_2015_237_MOESM6_ESM.docx]

| Age | Virus | Virus titers (EID50/g) | | | |
| --- | --- | --- | --- | --- | --- |
|  |  | Lung | | Spleen | |
|  |  | NDV | HPAIV | NDV | HPAIV |
| 2  weeks old | HPAIV | nd | 7.8/6.8 | nd | 7.1/6.2 |
|  | *m*NDV | -/- | nd | 2.8/- | nd |
|  | *m*NDV + HPAIV | -/- | 8.4/10.2 | -/- | 6.2/7.2 |
|  | *m*NDV + HPAIV 3 days later | 7.2/5.8 | 4.2/5.2 | 5.1/5.2 | 3.8/3.5 |
| 4  weeks old | HPAIV | nd | 7/8.1 | nd | 6.8/6.7 |
|  | *m*NDV | -/- | nd | 2.7/- | nd |
|  | *m*NDV + HPAIV | -/- | 9.4/4.9 | 5.4/4.4 | 5.4/3.5 |
|  | *m*NDV + HPAIV 3 days later | 6.6/6.5 | 4.5/4.9 | 5.6/5.6 | 3.3/4.4 |

nd = not done; - = negative
